# European Society of Pediatric Radiology survey of perioperative imaging in pediatric liver transplantation: (1) pre-transplant evaluation

**DOI:** 10.1007/s00247-023-05797-1

**Published:** 2023-11-21

**Authors:** Jochen Herrmann, Lil-Sofie Ording-Müller, Stéphanie Franchi-Abella, Martijn V. Verhagen, Simon P. McGuirk, Elena Dammann, Reinoud P. H. Bokkers, Philippe R. M. Clapuyt, Annamaria Deganello, Francesco Tandoi, Jean de Ville de Goyet, Hanna Hebelka, Charlotte de Lange, Cecile Lozach, Paolo Marra, Darius Mirza, Piotr Kalicinski, Janina M. Patsch, Giulia Perucca, Ilias Tsiflikas, Diane M. Renz, Bernd Schweiger, Marco Spada, Seema Toso, Loïc Viremouneix, Helen Woodley, Lutz Fischer, Philippe Petit, Florian Brinkert

**Affiliations:** 1 Section of Pediatric Radiology, Department of Diagnostic and Interventional Radiology and Nuclear Medicine, Universitatsklinikum Hamburg-Eppendorf, Martinistrasse 52, 20246 Hamburg, Germany; 2 Department of Pediatric Radiology, Oslo Universitetssykehus Rikshospitalet, Oslo, Norway; 3 Department of Pediatric Radiology, Hôpital Bicêtre, Paris, France; 4 Department of Radiology, University Medical Centre Groningen, Groningen, Netherlands; 5 Department of Radiology, Birmingham Children’s Hospital, Birmingham, UK; 6 Department of Radiology, Cliniques Universitaires Saint-Luc, Brussels, Belgium; 7 Department of Radiology, King’s College Hospital, London, UK; 8 Department of Hepatobiliary and Transplant Surgery, Azienda Ospedaliero-Universitaria Città Della Salute E Della Scienza Di Torino, Turin, Italy; 9 Department of Pediatrics and Pediatric Transplantation, ISMETT-UPMC, Palermo, Italy; 10 Department of Radiology, The Institute of Clinical Sciences, Gothenburg, Sweden; 11 Department of Pediatric Radiology, Queen Silvia Children’s Hospital, Gothenburg, Sweden; 12 Department of Radiology, Hôpital Universitaire Necker-Enfants-Malades, Paris, France; 13 Department of Radiology, Azienda Ospedaliera Ospedali Riuniti Di Bergamo: Aziende Socio Sanitarie Territoriale Papa Giovanni XXIII, Bergamo, Italy; 14 Department of Hepatobiliary and Transplant Surgery, Birmingham Children’s Hospital, Birmingham, UK; 15 Department of Pediatric Surgery and Organ Transplantation, The Children’s Memorial Health Institute, Warsaw, Poland; 16 Department of Radiology, Medical University of Vienna, Vienna, Austria; 17 Department of Radiology, Great Ormond Street Hospital for Children, London, UK; 18 Department of Pediatric Radiology, Regina Margherita Children’s Hospital, Turin, Italy; 19 Department of Radiology, University Clinic of Tübingen, Tübingen, Germany; 20 Department of Pediatric Radiology, Hannover Medical School Hospital, Hannover, Germany; 21 Institute of Diagnostic and Interventional Radiology and Neuroradiology, University Hospital Essen, Essen, Germany; 22 Division of Hepatobiliopancreatic Surgery, Liver and Kidney Transplantation, Ospedale Pediatrico Bambino Gesu, Rome, Italy; 23 Department of Pediatric Radiology, Geneva University Hospitals, Geneva, Switzerland; 24 Department of Radiology, Hôpital Femme Mère Enfant - Hospices Civils de Lyon, Bron, France; 25 Department of Pediatric Radiology, Leeds Children’s Hospital, Leeds, UK; 26 Department of Visceral Transplant Surgery, Universitatsklinikum Hamburg-Eppendorf, Hamburg, Germany; 27 Department of Pediatric Radiology, Hôpital de La Timone: Hopital de La Timone, Marseille, France; 28 Department of Pediatric Gastroenterology and Hepatology, University Clinic Hamburg-Eppendorf, Hamburg, Germany

**Keywords:** Child, Computed tomography, Liver transplantation, Magnetic resonance imaging, Ultrasonography

## Abstract

**Background:**

Liver transplantation is the state-of-the-art curative treatment in end-stage liver disease. Imaging is a key element for successful organ-transplantation to assist surgical planning. So far, only limited data regarding the best radiological approach to prepare children for liver transplantation is available.

**Objectives:**

In an attempt to harmonize imaging surrounding pediatric liver transplantation, the European Society of Pediatric Radiology (ESPR) Abdominal Taskforce initiated a survey addressing the current status of imaging including the pre-, intra-, and postoperative phase. This paper reports the responses on preoperative imaging.

**Material and methods:**

An online survey, initiated in 2021, asked European centers performing pediatric liver transplantation 48 questions about their imaging approach. In total, 26 centers were contacted and 22 institutions from 11 countries returned the survey. From 2018 to 2020, the participating centers collectively conducted 1,524 transplantations, with a median of 20 transplantations per center per annum (range, 8–60).

**Results:**

Most sites (64%) consider ultrasound their preferred modality to define anatomy and to plan surgery in children before liver transplantation, and additional cross-sectional imaging is only used to answer specific questions (computed tomography [CT], 90.9%; magnetic resonance imaging [MRI], 54.5%). One-third of centers (31.8%) rely primarily on CT for pre-transplant evaluation. Imaging protocols differed substantially regarding applied CT scan ranges, number of contrast phases (range 1–4 phases), and applied MRI techniques.

**Conclusion:**

Diagnostic imaging is generally used in the work-up of children before liver transplantation. Substantial differences were noted regarding choice of modalities and protocols. We have identified starting points for future optimization and harmonization of the imaging approach to multicenter studies.

**Supplementary Information:**

The online version contains supplementary material available at 10.1007/s00247-023-05797-1.

## Introduction

Liver transplantation is the state-of-the-art curative therapy for end-stage liver disease in children. Advances in organ procurement, surgical techniques, and immunosuppression have led to excellent short- and long-term results with a 5-year patient survival rate exceeding 85% [1–3]. Indications for liver transplantation in children differ from adults. Acquired cholestatic and genetic-metabolic disorders are the main underlying causes for pediatric liver transplantation, whereas end-stage liver disease due to viral hepatitis and hepatic malignancies, which represent major transplant reasons in adults, are rare [1].

Imaging methods are key elements for transplantation programs as they have been shown to assist surgical planning, to guide intraoperative surgical technique, and can be effectively applied to detect postoperative complications [4–9]. Preoperatively, the main objective for dedicated abdominal imaging is to measure organ and vessel sizes and depict vascular anatomy and patency, as well as to rule out contraindications potentially complicating surgery [4, 6, 10–12].

According to the European Association for the Study of the Liver (EASL) guidelines, it is mandatory to perform three-phase intravenous contrast computed tomography (CT) in adults to preoperatively evaluate the recipient anatomy. Contrast this to children, in whom ultrasound (US) is the main imaging technique due to its general availability, high spatial and temporal resolution, multiparametric capacity, and lack of radiation exposure [10, 11, 13]. While US exhibits relatively high accuracy in evaluating the pre-transplant abdominal status compared to intraoperative findings, CT and magnetic resonance imaging (MRI) are occasionally employed to offer greater detail and a reproducible roadmap for technically complex operations [12]. Nevertheless, these cross-sectional modalities present distinct advantages and disadvantages concerning availability, anatomical structure visualization, accuracy, and potential risks to the patient, such as anesthesia, examination duration, radiation exposure, and use of contrast agents [5, 12, 14].

So far, only limited data regarding the best imaging approach to prepare children for liver transplantation is available and is mostly based on expert opinion and small single center studies [4, 6, 10]. In an attempt to harmonize perioperative imaging for pediatric liver transplantation, the European Society of Pediatric Radiology (ESPR) Abdominal Taskforce initiated an online survey addressing common imaging practices at European centers including the pre-, intra-, and postoperative phase. This paper reports the responses on the preoperative imaging section of the survey in order to find a common basis for later consensus recommendations as well as for potential multicenter studies.

## Material and methods

### The survey

The online survey of the ESPR by the ESPR Abdominal Taskforce contacted European centers for pediatric liver transplantation asking about their current protocols regarding diagnostic imaging procedures. The survey followed a multidisciplinary approach, and the questions were directed towards all pediatric disciplines involved (e.g., radiology, transplantation surgery, gastroenterology, intensive care). A representative of each center was asked to gather the information from all sub-disciplines and to fill out the online survey using Google Forms. The survey was initiated in 2021 and the participating centers were asked to specify their liver transplantation numbers and choice of modalities in the period 2018−2020. A total of 48 questions were organized in six sections: demographics (seven questions), pre-transplant evaluation (eight questions), intraoperative imaging (eight questions), postoperative imaging (15 questions), liver elastography (six questions), and outlook (four questions). For the questions on pre-transplant evaluation, see Table 1. For the entire survey, see Supplementary Material 1.

**Table 1:** Survey details

1. Do you (as a representative of your center) agree that ultrasound (US) is the preferred imaging modality for definition of anatomy and to plan liver transplant surgery in children?	
	*yes/no*
2. Do you perform abdominal computed tomography (CT) imaging in children in preparation of liver transplantation?	
	*no/yes, in all patients/yes, in selected patients: In children *<*1 year, in children *<*6 years, in children not requiring sedation, in children with prior abdominal surgery (e.g., Kasai), in children with abnormalities on US, other: (free text)*
3. If yes, please specify your main indications for CT in pre-transplant evaluation:	
	*vascular anatomy/vascular patency/organ sizes/tissue and lesion characterization/other (free text)*
4. If yes, please specify the usual CT scan area:	
	*upper abdomen (liver)/complete abdomen*
5. If yes, please specify the CT phases you perform:	
	*native (no contrast)/arterial phase/parenchymal phase/late phase*
6. Do you perform abdominal magnetic resonance imaging (MRI) in children in preparation of liver transplantation?	
	*no/yes, in all patients/yes, in selected patients: In children *<*1 year, in children *<*6 years, in children not requiring sedation, in children with prior abdominal surgery (e.g., Kasai), in children with abnormalities on US, other: (free text)*
7. If yes, please check your main indications for MRI in pre-transplant evaluation:	
	*anatomy/vascular patency and flow/organ sizes/tissue and lesion characterization/other: (free text)*
8. If yes. Which MR vascular imaging technique do you use?	
	*Contrast-enhanced MR-Angiography (CE-MRA): 2-D/3-D/time resolved (4-D, temporal resolution below 5 seconds); Noncontrast MR-Angiography (NCE-MRA): Time of flight (TOF)/balanced steady state free precession (bSSFP: True FISP, Fiesta, b-FFE, B-Trance)/arterial spin labeling (ASL)/phase contrast angiography (PCA)/4-D Flow/other: (free text)*

*D* dimensional

## Results

### European sites (demographics)

**Fig. 1:**
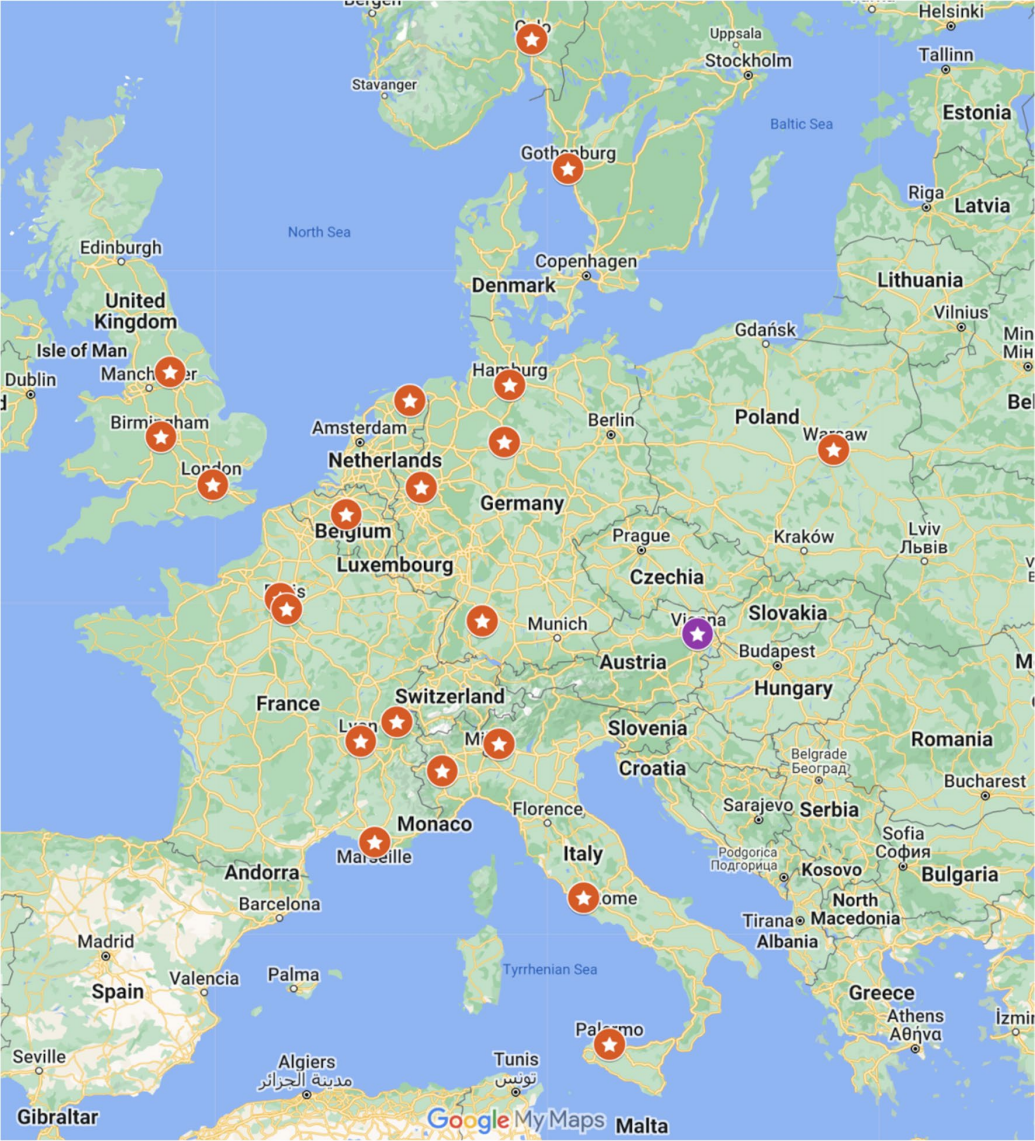
Participating European pediatric liver transplantation sites; one center (purple) only performed pre-operative evaluation and post-operative monitoring after discharge from the Intensive Care Unit. The liver transplantation was done at other sites

Twenty-six European centers known to our abdominal task force as liver transplantation centers were invited to participate in the survey, and 22 institutions returned the survey (survey return rate 84%). The centers are localized in 11 European countries and comprised 17 university children’s hospitals (77.3%), four specialist children’s hospitals (18.2%), and one district hospital (4.5%) (Fig. 1). Liver transplantation was performed in 21 centers. One center did not perform the liver transplantation and took over transplanted children from outside after discharge from intensive care, and thus omitted questions regarding intra-operative and early postoperative imaging.

During the period 2018–2020, a total of 1,524 pediatric liver transplantations were performed in the participating sites (median number per year and site, 20; range, 8–60). The proportions of transplantations performed per year in children <6 years and <1 year were 69.5% (353/508 liver transplantations) and 32.1% (163/508 liver transplantations), respectively. For children <6 years old, the median proportion of children at each center receiving split liver transplants was 87% (range, 40–100%), and 33.2% had living-related grafts (range, 1–90%).

### Ultrasound

**Fig. 2:**
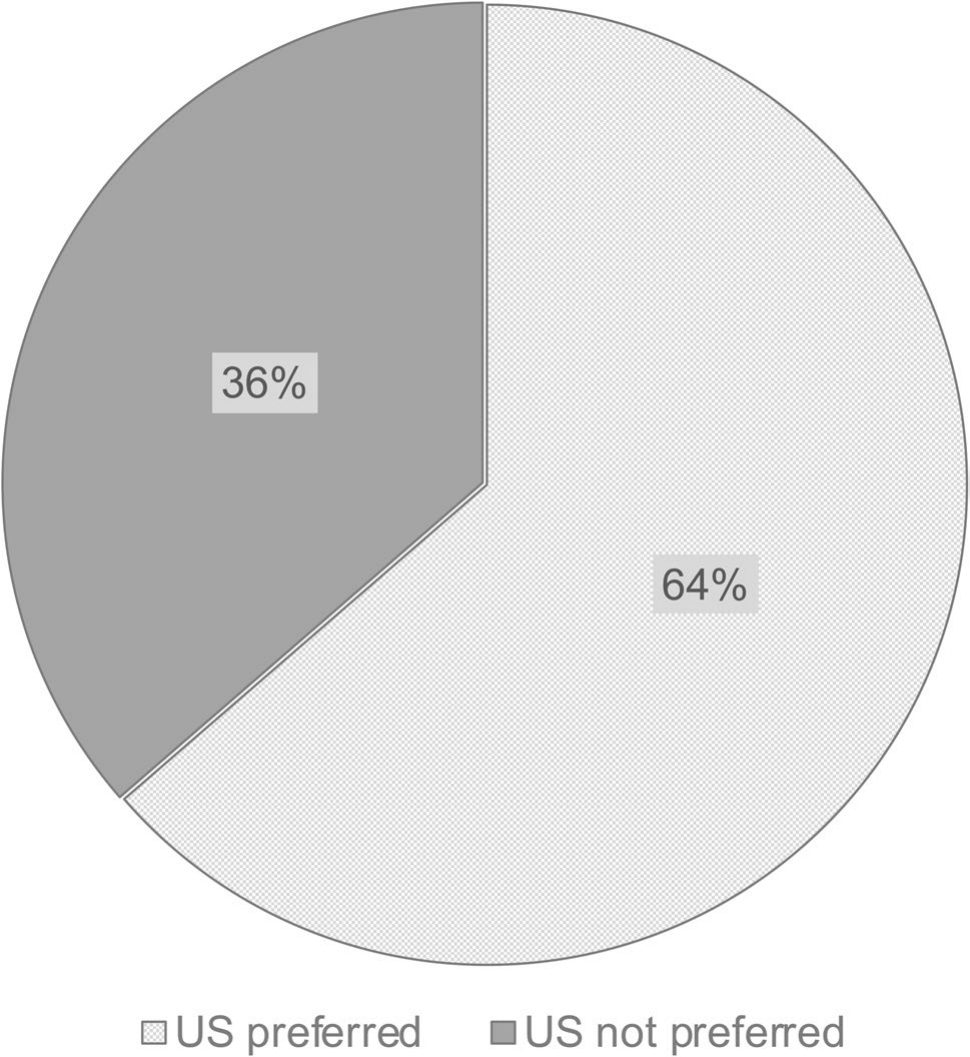
The role of ultrasound in defining anatomy and planning liver transplantation in children: Insights from 22 pediatric liver transplantation centers *US* ultrasound

All sites use abdominal US to assess children before liver transplantation. The majority of sites (14/22 sites, 64%) consider US as their preferred imaging modality to outline the anatomy and to plan surgery (Fig. 2). Additional cross-sectional imaging is only used by these sites in an attempt to answer specific questions in selected cases.

### Abdominal computed tomography and magnetic resonance imaging

**Fig. 3:**
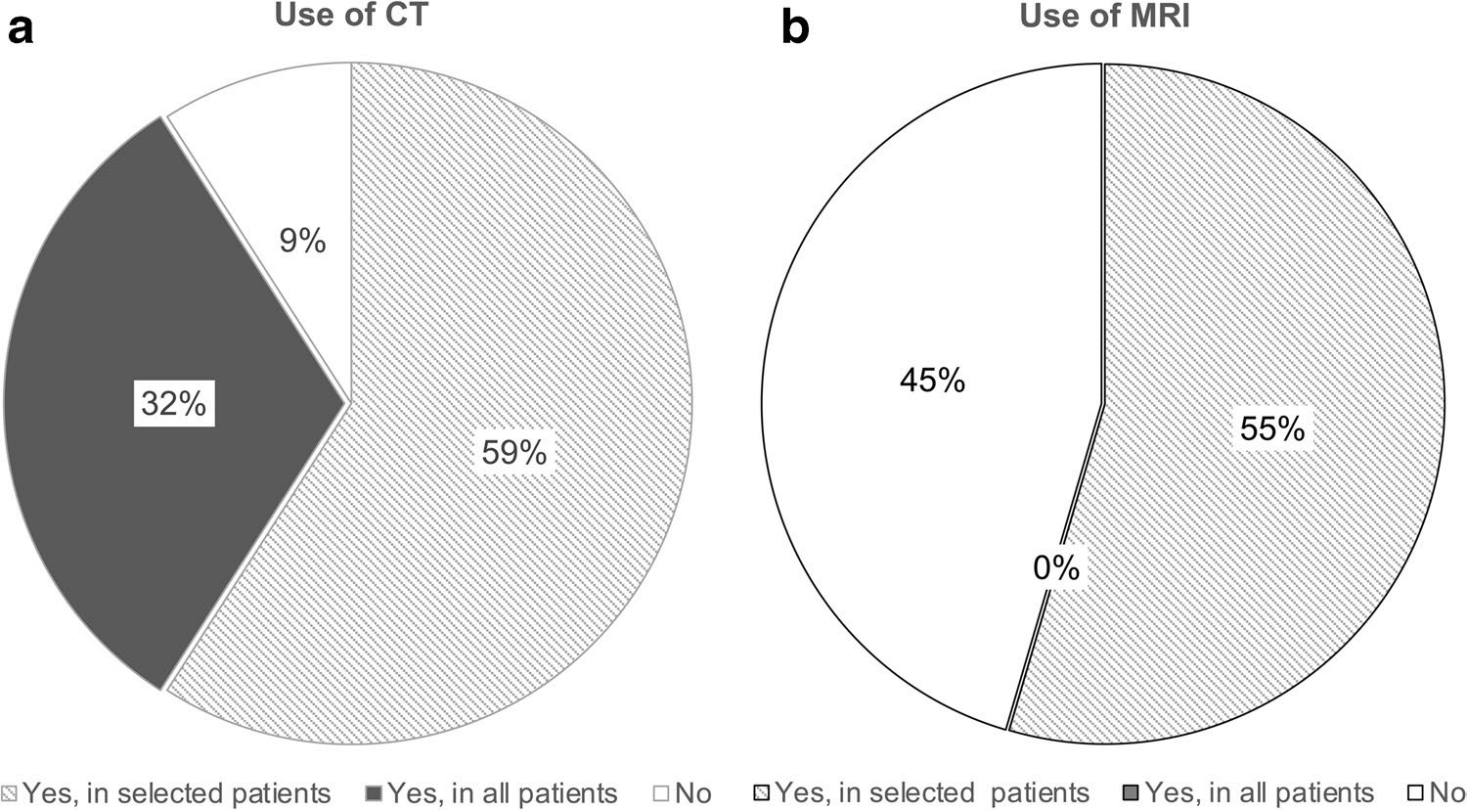
Use of abdominal computed tomography (**a**) and magnetic resonance imaging (**b**) in children in preparation for liver transplantation *CT* computed tomography, *MRI* magnetic resonance imaging

**Fig. 4:**
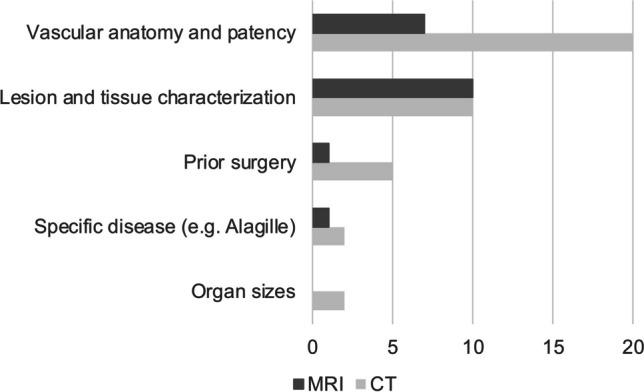
Indications for abdominal computed tomography (CT) and magnetic resonance imaging (MRI) in preparation for liver transplantation. Responses of 20 centers using CT and 12 centers using MRI

Cross-sectional imaging by CT or MRI is used by 20 of 22 sites (90.9%) in preparation for liver transplantation. Whereas the majority of sites perform abdominal CT only for selected cases when further clarification of abnormalities is needed, 7/22 sites (31.8%) always perform CT before liver transplantation (Fig. 3). The leading indications for abdominal CT are definition of the vascular anatomy and patency (20/20 sites, 100%), as well as lesion characterization including tumor restaging (6/13 centers, 46.2%) (Fig. 4). MRI is used for selected cases at the preoperative stage in 12 of 22 sites (54.5%). Only one center used MRI or CT in every patient. The main indications for abdominal MRI are lesion characterization (9/12, 75%) or the definition of vascular anatomy (7/12 centers, 58.3%) (Fig. 4). Abdominal MRI is also added in complicated cases, e.g., together with brain MRI in children needing sedation.

### Protocol differences

**Fig. 5:**
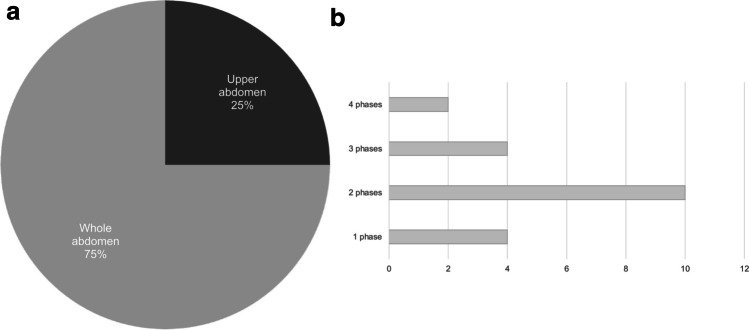
Scan range (**a**) and number of contrast phases applied (**b)** for abdominal computed tomography (CT) in children in preparation for liver transplantation. Responses of 20 sites using preoperative CT

**Fig. 6:**
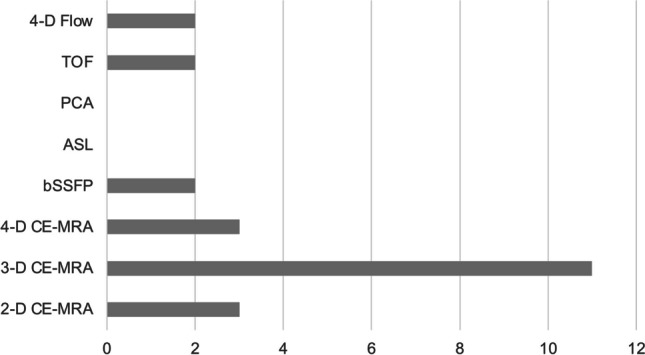
Magnetic resonance imaging (MRI) technique used to depict vascular anatomy and flow in preparation for liver transplantation. Responses of 12 sites using MRI diagnostics preoperatively. *ASL* arterial spin labeling, *bSSFP* balanced steady state free precession, *CE* contrast-enhanced, *D* dimensional, *MRA* MR-angiography, *PCA* phase contrast angiography, *TOF* time of flight

There is substantial variation among the participating centers in the approach to cross-sectional imaging. When CT is utilized for preoperative evaluation, most centers perform a complete abdominal scan, but in five of 20 institutions (25%), the scan area is limited to the upper abdomen. Additionally, there are differences in the number of contrast phases employed, ranging from a single- to a four-phase CT (Fig. 5). The majority of centers perform CT scan with two or more phases (80%, *n*=16). The preferred MRI technique for the assessment of vascular anatomy among most sites (11 out of 12, 91.7%) is dynamic contrast-enhanced 3-dimensional (D) MR-angiography (3-D MRA). A small number of institutions (3 out of 12, 25%) employ alternative methods such as 2-D MRA or high-temporal resolution 4-D MRA. Non-contrast MRI-based vascular imaging techniques, including 4-D-Flow, time of flight, and balanced steady state free precession, are rarely used (each at 2 out of 12 sites, 16.7%) as alternative approaches (Fig. 6).

## Discussion

This paper presents the preoperative imaging strategies derived from a multicenter European survey, which examines the current practices in pediatric liver transplantation imaging. The survey provides a comprehensive, multidisciplinary perspective on how imaging is integrated into the overall assessment of children with end-stage liver disease. Data were collected from 22 European centers, encompassing over 1,500 pediatric liver transplantations conducted over a 3-year period. As a result, the findings offer a representative overview of the prevailing imaging practices in this context. Details of the current use of imaging methods during the intra- and postoperative phase will be provided in dedicated publications of the results of this survey.

All sites use US to assess and monitor children before liver transplantation. Two-thirds of sites also consider US as their preferred and main imaging modality to outline anatomy and plan surgery. Especially in infants and lean children, Doppler US is feasible and highly accurate to detect arterial and venous abnormalities at the preoperative stage [12, 15]. The sites indicating US as their primary modality will only add further cross-sectional imaging if clarification is needed, e.g., to define vascular abnormalities suspected on US that may potentially complicate the transplantation, lesion characterization as part of oncologic staging, or prior surgery that increases the risk of unexpected findings.

The rather selective use of preoperative CT or MRI is explained by the high proportion of very young children qualifying for liver transplantation (70%<6 years of age), in keeping with previous publications [16]. Younger children have a higher sensitivity for the consequences of ionizing radiation [17–19]. In addition, depending on the CT machine and local preference, young children may require general anesthesia for CT. For MRI, the feed and wrap method may be used to avoid general anesthesia in children 0–6 months old, but anesthesia will generally be required between 6 months and 6 years [20, 21]. Nevertheless, one-third of the pediatric transplantation sites always perform contrast-enhanced CT in children awaiting liver transplantation. Their common indication is delineation of the child’s vascular anatomy, essentially representing the backbone for later transplantation. CT angiography can depict vascular anatomy also in young children with very high accuracy and may be the best method to show subtle hepatic artery anomalies [5, 14].

This survey revealed substantial differences regarding the basic CT scan parameters used at the centers. The majority of centers imaged the entire abdomen, whereas a quarter limited their CT scans to the upper abdomen only. The number of contrast phases usually applied at each center varied between one and four. This suggests the potential for further dose saving. A split bolus technique, for example, generates high contrast images of all hepatic vessels in one CT phase, omitting the need for additional acquisitions [22, 23]. This technique has also been evaluated in the context of pediatric trauma, oncologic disease for characterization of focal liver lesions in adults, and renal donor evaluation [24–26].

Abdominal MRI, which is a radiation-free alternative to CT, is regularly used by those transplantation sites not primarily relying on CT at the preoperative stage. The most frequent indication is lesion characterization for which hepatic MRI is the method of choice, offering dynamic imaging and liver-specific contrast agents (in some countries off-label) [27–30]. Compared to CT, fewer centers use abdominal MRI to image vascular anatomy. The most applied technique is 3-DMRA which may show slightly inferior accuracy to CT angiography for depiction of arterial anatomy as suggested by adult and a few pediatric studies [5, 14, 31]. However, with newer MRI techniques (e.g., 3-tesla MRI, time-resolved contrast-enhanced MRA), the state-of-the-art techniques may change soon and should be the object of future comparative studies [14].

## Conclusion

The survey findings indicate that medical imaging plays an essential role in the pre-transplant evaluation of children across all participating centers. Whereas US is the preferred modality at most European sites and additional cross-sectional imaging is only used in selected cases, some centers always opt for CT to obtain a detailed anatomical map before liver transplantation. As an outline for future directions, it is important to explore whether acquisition of CT data really is required as a standard pre-transplant examination in children or whether all transplant centers ideally should replace this examination with US or MRI and reserve CT for selected cases*.* Furthermore, significant variations in CT and MRI protocols were observed, suggesting the potential for optimization. The development of a structured and harmonized approach to pre-transplant imaging in children is crucial for future multicenter studies, where imaging parameters can serve as primary endpoints.

### Supplementary Information

ESM 1 (PDF 139 kb)

## Data Availability

The datasets generated during and analyzed during the current survey research are not publicly available as individual privacy was guaranteed to all participating centers. Blinded data are however available from the authors upon reasonable request and with permission of all participating centers.
