# Correction to: European Society of Pediatric Radiology survey of perioperative imaging in pediatric liver transplantation: (1) pre‑transplant evaluation

**DOI:** 10.1007/s00247-023-05843-y

**Published:** 2024-01-09

**Authors:** Jochen Herrmann, Lil‑Sofie Ording‑Müller, Stéphanie Franchi‑Abella, Martijn V. Verhagen, Simon P. McGuirk, Elena Dammann, Reinoud P. H. Bokkers, Philippe R. M. Clapuyt, Annamaria Deganello, Francesco Tandoi, Jean de Ville de Goyet, Hanna Hebelka, Charlotte de Lange, Cecile Lozach, Paolo Marra, Darius Mirza, Piotr Kalicinski, Janina M. Patsch, Giulia Perucca, Ilias Tsiflikas, Diane M. Renz, Bernd Schweiger, Marco Spada, Seema Toso, Loïc Viremouneix, Helen Woodley, Lutz Fischer, Philippe Petit, Florian Brinkert

**Affiliations:** 1 Section of Pediatric Radiology, Department of Diagnostic and Interventional Radiology and Nuclear Medicine, Universitatsklinikum Hamburg-Eppendorf, Martinistrasse 52, 20246 Hamburg, Germany; 2 Department of Pediatric Radiology, Oslo Universitetssykehus Rikshospitalet, Oslo, Norway; 3 Department of Pediatric Radiology, Hôpital Bicêtre, Paris, France; 4 Department of Radiology, University Medical Centre Groningen, Groningen, Netherlands; 5 Department of Radiology, Birmingham Children’s Hospital, Birmingham, UK; 6 Department of Radiology, Cliniques Universitaires Saint-Luc, Brussels, Belgium; 7 Department of Radiology, King’s College Hospital, London, UK; 8 Department of Hepatobiliary and Transplant Surgery, Azienda Ospedaliero-Universitaria Città Della Salute E Della Scienza Di Torino, Turin, Italy; 9 Department of Pediatrics and Pediatric Transplantation, ISMETT-UPMC, Palermo, Italy; 10 Department of Radiology, The Institute of Clinical Sciences, Gothenburg, Sweden; 11 Department of Pediatric Radiology, Queen Silvia Children’s Hospital, Gothenburg, Sweden; 12 Department of Radiology, Hôpital Universitaire Necker-Enfants-Malades, Paris, France; 13 Department of Radiology, Azienda Ospedaliera Ospedali Riuniti Di Bergamo: Aziende Socio Sanitarie Territoriale Papa Giovanni XXIII, Bergamo, Italy; 14 Department of Hepatobiliary and Transplant Surgery, Birmingham Children’s Hospital, Birmingham, UK; 15 Department of Pediatric Surgery and Organ Transplantation, The Children’s Memorial Health Institute, Warsaw, Poland; 16 Department of Radiology, Medical University of Vienna, Vienna, Austria; 17 Department of Radiology, Great Ormond Street Hospital for Children, London, UK; 18 Department of Pediatric Radiology, Regina Margherita Children’s Hospital, Turin, Italy; 19 Department of Radiology, University Clinic of Tübingen, Tübingen, Germany; 20 Department of Pediatric Radiology, Hannover Medical School Hospital, Hannover, Germany; 21 Institute of Diagnostic and Interventional Radiology and Neuroradiology, University Hospital Essen, Essen, Germany; 22 Division of Hepatobiliopancreatic Surgery, Liver and Kidney Transplantation, Ospedale Pediatrico Bambino Gesu, Rome, Italy; 23 Department of Pediatric Radiology, Geneva University Hospitals, Geneva, Switzerland; 24 Department of Radiology, Hôpital Femme Mère Enfant - Hospices Civils de Lyon, Bron, France; 25 Department of Pediatric Radiology, Leeds Children’s Hospital, Leeds, UK; 26 Department of Visceral Transplant Surgery, Universitatsklinikum Hamburg-Eppendorf, Hamburg, Germany; 27 Department of Pediatric Radiology, Hôpital de La TimoneHopital de La Timone, Marseille, France; 28 Department of Pediatric Gastroenterology and Hepatology, University Clinic Hamburg-Eppendorf, Hamburg, Germany

**Correction to: Pediatric Radiology (2023)**

https://doi.org/ 10.1007/s00247-023-05797-1

The originally published article contains errors.

1.  In the Abstract, the original published online version stated,

“An online survey, initiated in 2021, asked European centers performing pediatric liver transplantation 44 questions about their imaging approach.”

This was an error and the sentence should read,

“An online survey, initiated in 2021, asked European centers performing pediatric liver transplantation 48 questions about their imaging approach.”

2. In Materials and methods, the original published online version stated,

"A total of 44 questions were organized in six sections: demographics (seven questions), pre-transplant evaluation (eight questions), intraoperative imaging (eight questions), postoperative imaging (11 questions), liver elastography (six questions), and outlook (four questions)."

This was an error, and the sentence should read,

"A total of 48 questions were organized in six sections: demographics (seven questions), pre-transplant evaluation (eight questions), intraoperative imaging (eight questions), postoperative imaging (15 questions), liver elastography (six questions), and outlook (four questions)."

3. In Materials and methods, the original published online version stated,

"For the questions on pre-transplant evaluation, see Table 1."

This was an error, and the sentence should read,

"For the questions on pre-transplant evaluation, see Table 1. For the entire survey, see Supplementary Material 1"

4. In the published online version, the original caption for Figure 3 stated,

"*MRI* magnetic resonance imagin0"

This was an error, and the caption should read,

"*MRI* magnetic resonance imaging"

5. In Results, the original published online version used the abbreviations,

 “2-DMRA”, “3-DMRA” and “4-DMRA”,

This was an error, and the abbreviations should read,

“2-D MRA”, "3-D MRA” and “4-D MRA."

The original article has been corrected.

